# A case of reflective evidence-based surgery

**DOI:** 10.1308/003588413X13511609956417

**Published:** 2013-01

**Authors:** D Nikkhah

**Affiliations:** University College London,UK

A fellow colleague in the emergency department consulted me for advice regarding a two-year-old child with a facial laceration. The child had had a simple fall against a wooden table and sustained a 3cm clean linear laceration in the intercanthal area. My colleague asked me whether glue would be a better idea than suturing. I replied that the cosmetic outcome with suturing was far superior and that one would avoid the risk of dehiscence. My reply was based on anecdotal evidence from other colleagues and senior surgeons. This doctor followed my advice and the child had her wound sutured under general anaesthesia with no complications.

I later thought to myself that there should be a body of evidence to support or refute my advice on this particular issue. I knew that strong evidence such as a randomised controlled trial (RCT) would answer this question. I performed a MEDLINE^®^ search expecting a paucity of evidence; instead I was greeted with a Cochrane review and a number of prospective RCTs.^1^ I discovered that suturing conferred no benefit in terms of cosmesis in the paediatric population but that there was a statistically significant increased risk of dehiscence. This level 1 evidence has changed my practice. With hindsight I would explain this evidence to the parents and offer the option of glue. Indeed, the glue technique would obviate the risks of general anaesthesia and a hospital stay.

As surgeons, we can only be effective if we question our practice daily. We should always endeavour to practise research to strive for the truth as this will improve patient care. Evidence-based practice can save our hospitals money and time, particularly in a period of such financial upheaval.
