# Erratum

**Published:** 2013-03

*Ann R Coll Surg Engl* 2013; **95**: 83

**A case of reflective evidence-based surgery**

**D Nikkhah**

The fourth sentence in the second paragraph should read: ‘I discovered that suturing conferred no benefit in terms of cosmesis in the paediatric population but that there was a small but statistically significant *decreased* risk of dehiscence.’ We apologise for any confusion.

